# Diagnostic and therapeutic challenges of diffuse abdominal infantile hemangioma: a case report emphasizing multimodality imaging and multidisciplinary management

**DOI:** 10.3389/fped.2025.1738363

**Published:** 2026-01-07

**Authors:** Yan Yang, Minggang Yi, Jianshe Zhao

**Affiliations:** Department of Radiology, Jinan Children’s Hospital, Jinan, China

**Keywords:** diffuse abdominal infantile hemangioma, multidisciplinary management, multimodality imaging, propranolol, pulmonary hypertension

## Abstract

**Background:**

Diffuse abdominal infantile hemangioma (DAIH) is an exceptionally rare and life-threatening vascular anomaly, characterized by extensive infiltrative growth. It poses significant diagnostic challenges by mimicking malignancy and therapeutic difficulties due to involvement of critical structures. This case highlights the pivotal role of multimodality imaging and a multidisciplinary approach in its management.

**Case description:**

An 8-month-old female infant presented with abdominal distension and a history of hematochezia. Multimodality imaging (ultrasound, CT, and MRI) revealed an extensive, infiltrative vascular mass involving the mesentery and small bowel wall. Key findings included marked arterial enhancement, persistent venous pooling, flow voids, and secondary hemodynamic changes (portal/hepatic vein dilatation, aortic narrowing, and pulmonary hypertension). Given the lesion's unresectability and high embolization risk, ultrasound-guided biopsy was performed, confirming GLUT-1 positive infantile hemangioma. Oral propranolol therapy was initiated, leading to a dramatic reduction in lesion size and vascularity on follow-up imaging, along with resolution of pulmonary hypertension.

**Conclusions:**

This case underscores the characteristic imaging spectrum of DAIH, which radiologists must recognize to avoid misdiagnosis as malignancy. It reaffirms that a multidisciplinary strategy—centered on imaging-guided biopsy and propranolol as first-line therapy—can optimize outcomes even in severe cases, establishing a modern standard of care for such complex vascular anomalies.

## 1 Introduction

Infantile hemangiomas (IHs) are the most common benign vascular tumors of infancy (1). Although most are cutaneous and self-limiting, they can also occur in visceral organs, with the liver being the most frequent extracutaneous site (2). Diffuse vascular lesions originating from the mesentery are even rarer. Diffuse abdominal infantile hemangioma (DAIH), considered part of the diffuse neonatal haemangiomatosis (DNH) spectrum, is an exceptionally rare and aggressive subtype with a historically poor prognosis (3). Reported cases are scarce, often within larger series on visceral hemangiomas or DNH, underscoring its diagnostic and therapeutic challenge (3, 4). Its clinical and imaging presentation can closely mimic abdominal malignancies such as neuroblastoma or sarcoma, creating a significant diagnostic challenge.

Imaging plays a critical role in initial evaluation and differential diagnosis. Ultrasound with Doppler typically shows a heterogeneous, hypervascular mass. Cross-sectional imaging with contrast-enhanced computed tomography (CT) and magnetic resonance imaging (MRI) can characterize the lesion's intense enhancement and infiltrative growth pattern along the mesentery and bowel wall, which are key diagnostic features (5). Furthermore, high-flow shunting within these extensive lesions can lead to secondary systemic complications, including high-output cardiac failure and pulmonary hypertension (6).

The management of DAIH is complex. Due to the diffuse involvement of vital abdominal structures, complete surgical resection is often not feasible (3), while transarterial embolization carries a substantial risk of bowel ischemia and necrosis. Therefore, a multidisciplinary approach is imperative. A diagnostic strategy involving biopsy for pathological confirmation followed by medical therapy with propranolol has proven successful (2, 7). We present a detailed case of DAIH to highlight the indispensable role of multimodality imaging in diagnosis, biopsy guidance, complication assessment, and treatment monitoring.

## 2 Case information

An 8-month-old female infant was referred for evaluation of a large abdominal mass, which was initially discovered during a prior hospitalization for hematochezia at one month of age. Her past medical history was notable for neonatal necrotizing enterocolitis and septic shock. Physical examination revealed a markedly distended, soft, and non-tender abdomen, with palpable hepatomegaly.

**Figure 1:**
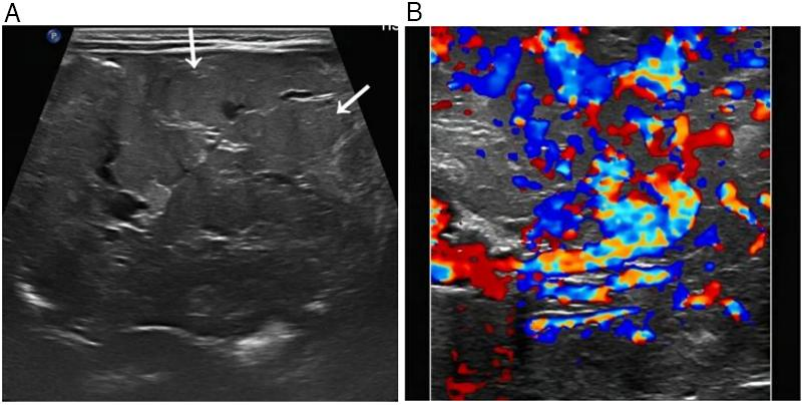
Abdominal ultrasound. **(A)** Grayscale image shows an extensive, ill-defined, heterogeneous mass in the mesentery**(→)**. **(B)** Color Doppler image reveals rich, chaotic vascularity within the mass.

**Figure 2:**
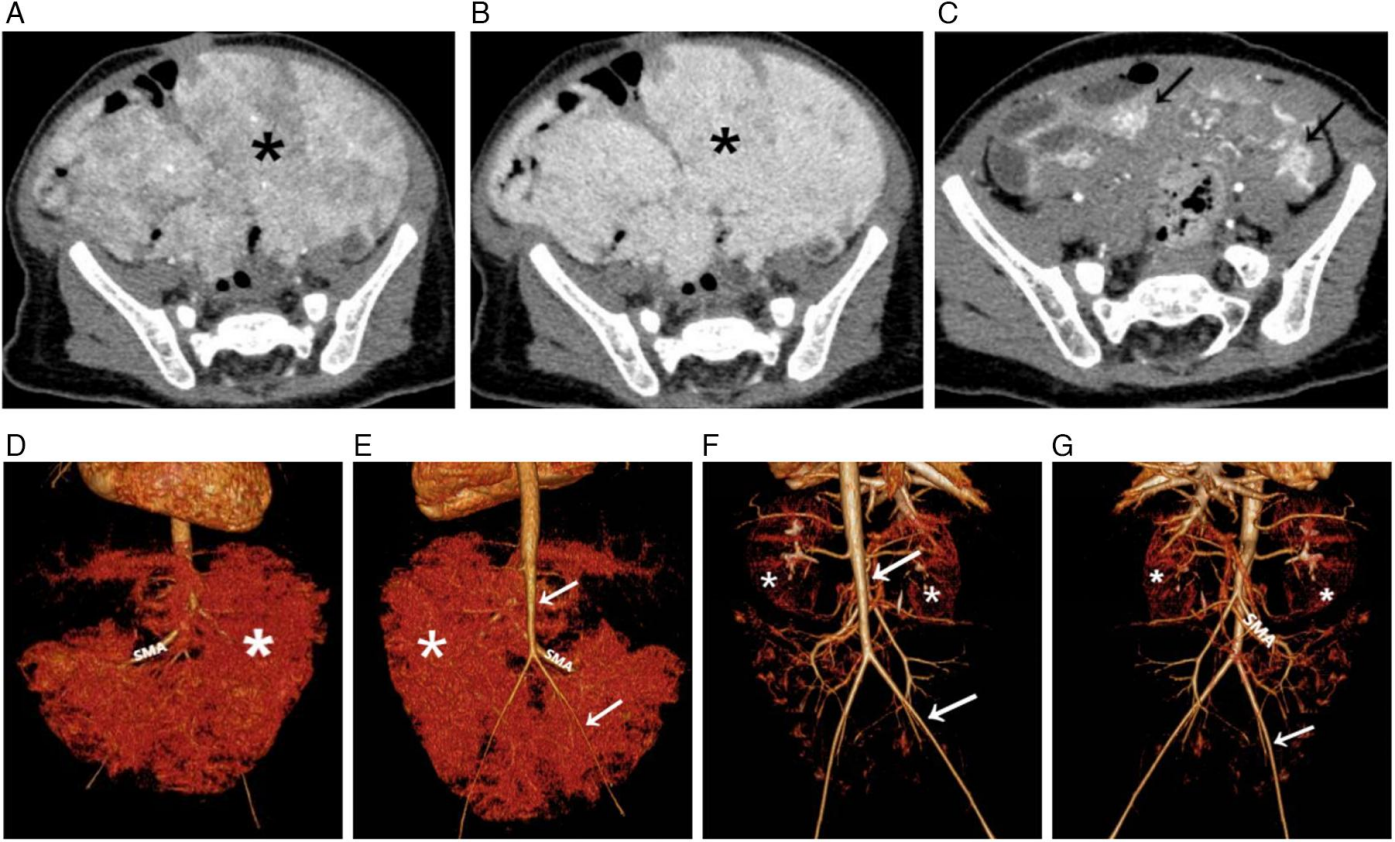
Contrast-enhanced CT, before and after treatment. **(A,B)** Pre-treatment axial CT images show a massive infiltrative mass (*) encasing mesenteric vessels and bowel loops, exhibiting heterogeneous arterial phase enhancement **(A)** and persistent, near-homogeneous venous phase enhancement **(B,C)** Post-treatment axial CT image reveals marked regression of the mass with only residual patchy enhancement (→). **(D,E)** Pre-treatment volume-rendered (VR) CT images depict the large hypervascular mass (*), a markedly dilated superior mesenteric artery (SMA), and significant narrowing of the distal aorta (→). **(F,G)** Post-treatment VR images confirm mass regression (*) and mild improvement in the previously noted arterial narrowing (→).

**Figure 3:**
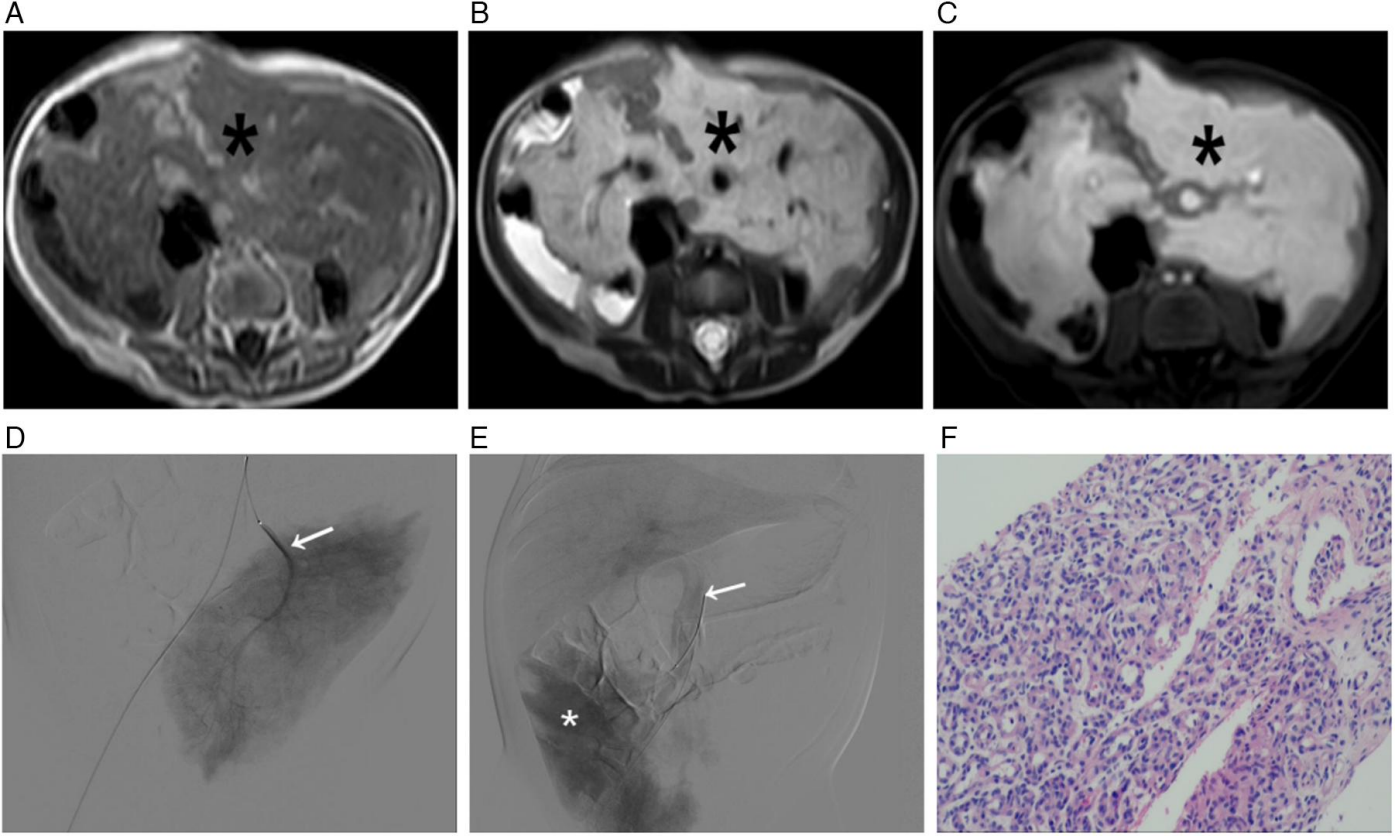
Multimodality characterization and pathological correlation of the mass. **(A–C)** MRI Features **(A)** Axial T1-weighted image shows the mass (*) as mildly hypointense. **(B)** Axial T2-weighted imaging demonstrates marked T2 hyperintensity (*). **(C)** Axial contrast-enhanced T1-weighted image reveals progressive, intense enhancement (*). **(D,E)** Digital Subtraction Angiography (DSA) **(D)** Arterial phase frontal projection shows the mass supplied by hypertrophied branches of the superior mesenteric artery (SMA) (→). **(E)** Substantive phase image reveals an intense, persistent tumor blush (*) with early opacification of the dilated portal venous system (→), confirming high-flow shunting. **(F)** Photomicrograph (H&E stain) of the biopsy specimen shows proliferating capillaries lined by plump endothelial cells.

A comprehensive multimodality imaging workup was critical for lesion characterization. Abdominal ultrasound revealed an extensive, ill-defined, heterogeneous mesenteric mass extending to the left flank and pelvis, appearing as a conglomerate thickening with numerous tortuous anechoic vascular channels (Figure 1A). Color Doppler imaging demonstrated profuse, chaotic vascular flow (Figure 1B). Secondary hemodynamic alterations included dilation of the proximal superior mesenteric vein, portal vein, hepatic veins, and intrahepatic inferior vena cava. Contrast-enhanced CT delineated a massive, infiltrative soft-tissue mass enveloping mesenteric vessels and bowel loops. It showed intense, heterogeneous arterial-phase enhancement (Figure 2A) and persistent, near-homogeneous venous-phase enhancement (Figure 2B). Notable vascular alterations included marked dilation of the superior mesenteric artery (SMA) with consequent narrowing of the distal aorta and its major branches, splenic vein compression, and significant portal venous system dilation. Volume-rendered CT images clearly depicted the hypervascular mass, dilated SMA, and narrowed distal aorta (Figures 2D,E). MRI confirmed extensive, confluent lesions along the mesenteric border of the bowel. The mass was mildly hypointense on T1-weighted imaging (Figure 3A) and markedly hyperintense on T2-weighted imaging (Figure 3B). Dynamic contrast-enhanced MRI showed marked progressive enhancement (Figure 3C), corroborating the CT findings. The mass encased the superior and inferior mesenteric vessels, accompanied by venous dilation and aortic narrowing. Localized intestinal wall thickening indicated direct bowel involvement.

Digital subtraction angiography (DSA) performed during the biopsy procedure confirmed the mass was supplied by hypertrophied SMA branches, revealing an intense, persistent tumor blush with early opacification of dilated draining veins and the portal system (Figures 3D,E).

Echocardiography revealed significant sequelae of the high-output state, including secondary pulmonary hypertension (estimated pulmonary artery systolic pressure 78 mmHg), right heart dilation, and moderate-to-severe tricuspid regurgitation. A secundum atrial septal defect and an aberrant right subclavian artery were also noted.

Given the diffuse, infiltrative nature of the mass involving the mesentery and bowel wall, the multidisciplinary team consensus deemed complete surgical resection impossible and transarterial embolization prohibitively risky due to the potential for bowel ischemia. Consequently, an ultrasound-guided percutaneous core needle biopsy was performed using an 18-gauge needle, obtaining five tissue cores from the mass. The procedure was uncomplicated. Postoperatively, the patient remained hemodynamically stable with no fever, rash, bleeding, or gastrointestinal discomfort. However, on the evening of the procedure, she developed tachypnea and dyspnea, which stabilized after supplemental oxygen via face mask and intravenous furosemide administration.

Histopathological examination of the biopsy specimens revealed proliferating capillaries lined by plump endothelial cells, admixed with a small amount of striated muscle, consistent with a vascular tumor (Figure 3F). The diagnosis of infantile hemangioma was confirmed by immunohistochemistry, which showed positive staining for CD31, CD34, GLUT-1, SMA, and Ki-67 (approximately 10%), while being negative for D2-40, PROX1, and Desmin (Figure 3F).

Based on the diagnosis, treatment with oral propranolol was initiated under cardiac monitoring. The dosage was titrated as follows: 0.5 mg/kg/day (0.5 mL) on day one, increased to 1 mg/kg/day (1 mL) on day two, and further to 1.9 mg/kg/day (1.9 mL) on day three. At discharge, the regimen included propranolol oral solution (initially 1.9 mL, later increased to 2.3 mL twice daily), hydrochlorothiazide (7.5 mg twice daily), and spironolactone (7.5 mg twice daily). The patient was followed up monthly for the first three months, then every 2–3 months thereafter, with regular ultrasonography or CT, echocardiography, and laboratory tests to monitor treatment response and safety.

At the six-month follow-up, the patient showed marked clinical and radiological improvement. Abdominal distension had substantially resolved. Follow-up echocardiography demonstrated significant resolution of the secondary pulmonary hypertension to normal pressures, with the associated tricuspid regurgitation improving to only a mild degree. Follow-up imaging confirmed dramatic regression of the mass and its vascularity, with only residual patchy enhancement on CT (Figure 2C) and mild improvement in the previously noted arterial narrowing on volume-rendered images (Figures 2F,G). Throughout the treatment period, the patient remained in good general condition with normal growth and no significant gastrointestinal symptoms.

## 3 Discussion

This case of diffuse abdominal infantile hemangioma (DAIH) exemplifies a significant diagnostic and therapeutic challenge in pediatric medicine (3). Its radiological features can closely mimic those of malignant abdominal tumors, potentially leading to unnecessary and aggressive interventions. Although the imaging characteristics were suggestive of a vascular anomaly, the initial presentation raised legitimate concerns for more common malignancies. Specifically, the infiltrative growth pattern and heterogeneous enhancement warranted consideration of infantile fibrosarcoma. However, the absence of a dominant solid necrotic component and the presence of characteristic progressive centripetal contrast fill-in on delayed phases provided crucial imaging clues favoring DAIH (6). Neuroblastoma constituted another key differential diagnosis, but the lack of calcifications on CT, the pattern of diffuse mesenteric and bowel wall involvement, and normal catecholamine levels effectively argued against this possibility (4).

The imaging hallmarks of DAIH across modalities are critical for accurate diagnosis. Ultrasonography typically reveals an ill-defined, heterogeneous mass with profuse vascularity on Doppler. Cross-sectional imaging with CT and MRI best demonstrates the lesion's infiltrative nature along the mesentery and bowel wall, characterized by intense arterial enhancement with persistent venous pooling, flow voids, and secondary vascular alterations (e.g., mesenteric artery dilation and aortic narrowing). MRI further aids characterization by showing T2 hyperintensity and progressive enhancement. DSA, while not always necessary for diagnosis, definitively illustrates the angioarchitecture, confirming high-flow shunting with early venous drainage.

The observed profound secondary hemodynamic alterations—including the “mesenteric steal” phenomenon, significant dilatation of the portal and hepatic venous systems, and the consequent high-output cardiac failure and pulmonary hypertension—vividly illustrate the substantial systemic burden imposed by this high-flow vascular condition (2, 8). These consequences serve as objective indicators of disease severity and significantly constrain therapeutic options.

Confronted with this diffuse, life-threatening lesion, our multidisciplinary team consensus against primary surgical resection or transarterial embolization reflects a modern, risk-averse therapeutic strategy. Surgical intervention carries an unacceptable risk of short bowel syndrome and uncontrollable hemorrhage, while embolization is fraught with the peril of extensive bowel necrosis. This therapeutic dilemma highlights the indispensable role of image-guided percutaneous core needle biopsy in obtaining a definitive histopathological diagnosis. The confirmation of GLUT-1 immunopositivity was a pivotal turning point, pathognomonically confirming infantile hemangioma and enabling a paradigm shift towards targeted, low-risk medical management (9).

Following oral propranolol therapy, the patient demonstrated rapid clinical and imaging improvement, consistent with its established role as a first-line treatment for infantile hemangioma (7). This agent offers a favorable safety profile and the convenience of oral administration. In cases resembling DNH or those complicated by high-output cardiac failure, propranolol has shown efficacy in reducing tumor volume and alleviating hemodynamic complications, including pulmonary hypertension (2, 10).

Nevertheless, not all visceral or diffuse hemangiomas respond adequately to propranolol. Alternative options may be considered for patients with intolerance or suboptimal response. Corticosteroids can be used in propranolol-intolerant individuals; however, their utility is limited by systemic side effects and uncertain efficacy in intestinal lesions (3). Interferon-α and vinca alkaloids (e.g., vincristine) have been employed in refractory cases. Vincristine has shown some benefit in steroid-resistant or life-threatening vascular anomalies such as Kasabach-Merritt syndrome and hepatic hemangioendothelioma (11). However, interferon carries a risk of neurotoxicity (e.g., spastic diplegia), vinca alkaloids are cytotoxic and unsuitable for long-term use in infants, and both agents may encounter resistance (3, 12–14). Sirolimus, an mTOR inhibitor, has emerged as a promising second-line option, particularly for propranolol-resistant diffuse lesions or hemangiomas associated with PHACE syndrome. Its mechanism involves anti-angiogenic effects via mTOR pathway inhibition, though further pediatric studies are warranted for validation (12). Surgical resection or embolization is generally reserved for localized, symptomatic lesions or acute complications such as perforation, as extensive intervention in diffuse disease carries significant risks of intestinal ischemia or short-bowel syndrome (4, 15).

The successful stabilization of this critically ill patient with oral propranolol reinforces the contemporary principle that even severe infantile hemangiomas represent medically manageable conditions (1, 2, 7, 10, 14).

This case offers several key teaching points:

▪ Diagnostic precision: DAIH can mimic malignancy, but key radiological differentiators include its intense enhancement, flow voids, and characteristic mesenteric/bowel wall infiltration, which should steer the diagnosis towards a vascular anomaly.
▪ Management strategy: A multidisciplinary approach is paramount. When resection or embolization is too risky, image-guided biopsy for GLUT-1 confirmation provides a safe pathway to a definitive diagnosis.
▪ Therapeutic efficacy: Propranolol monotherapy can be a highly effective first-line treatment, even in severe, life-threatening DAIH with systemic complications, leading to dramatic regression.

## 4 Conclusion

In conclusion, this case highlights the distinctive imaging features and multidisciplinary management challenges of diffuse abdominal infantile hemangioma (DAIH). Radiologists must recognize its characteristic—though occasionally deceptive—imaging findings to ensure accurate diagnosis and avoid misclassification as malignancy. The successful outcome in this high-risk patient underscores the critical importance of a multidisciplinary strategy, the diagnostic safety and efficacy of image-guided biopsy, and the potent efficacy of propranolol as first-line therapy. This integrated management approach optimizes patient outcomes and should be regarded as the standard of care for similar complex vascular anomalies.

## Data Availability

The original contributions presented in the study are included in the article/Supplementary Material, further inquiries can be directed to the corresponding author.
