# Supplementary material for: Diagnostic and therapeutic challenges of diffuse abdominal infantile hemangioma: a case report emphasizing multimodality imaging and multidisciplinary management
Source: Front Pediatr. 2026 Jan 7;13:1738363. doi: 10.3389/fped.2025.1738363 (PMC12819742; doi:10.3389/fped.2025.1738363)
Supplement: Supplementary file 1 [file Supplementaryfile1.docx]

Supplementary Material

FIGURE


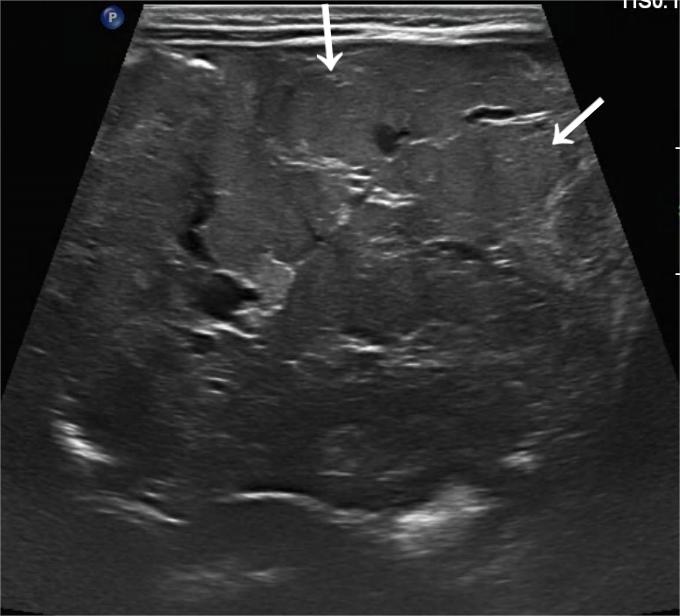

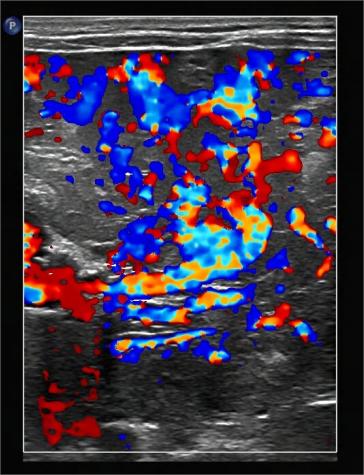


**1 A 1B**

**Fig. 1 Abdominal Ultrasound.
A** Grayscale image shows an extensive, ill-defined, heterogeneous mass in the mesentery(→). **B** Color Doppler image reveals rich, chaotic vascularity within the mass.


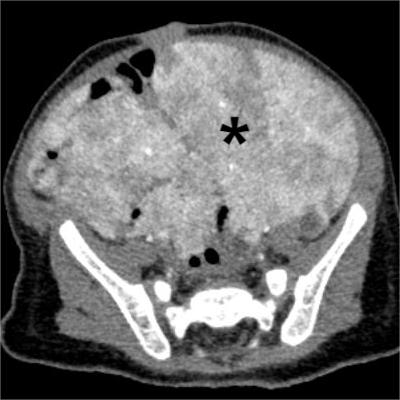

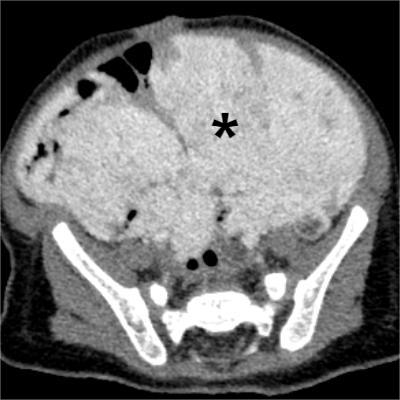

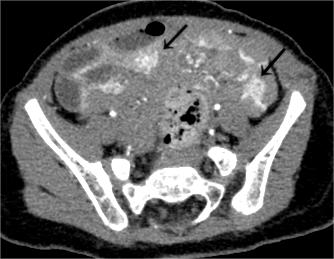


**2A 2B 2C**


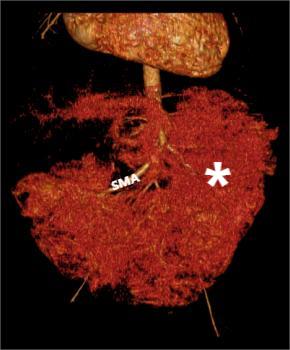

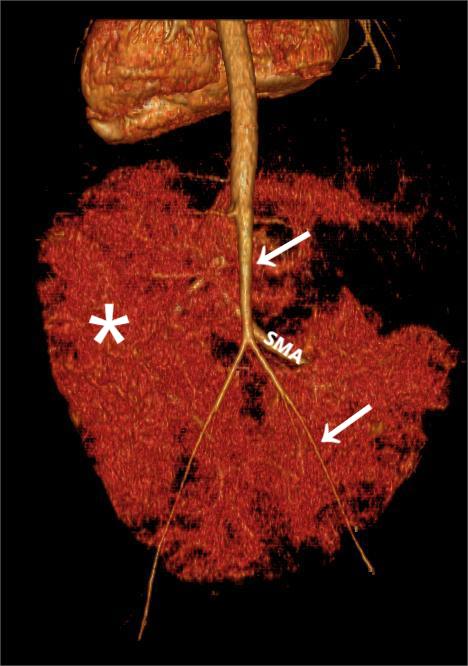

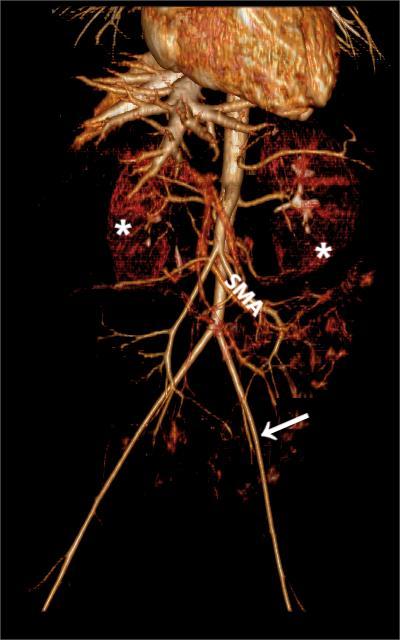

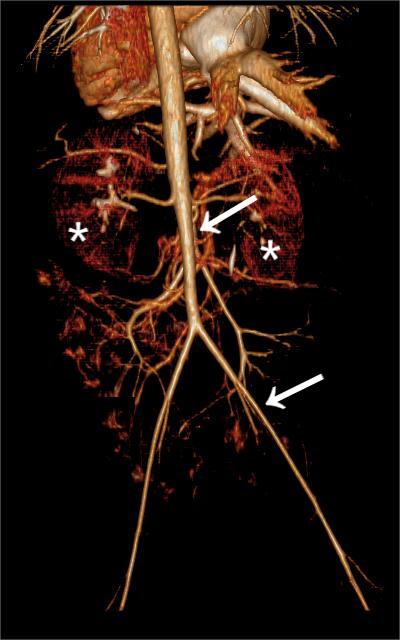


**2D 2E 2F 2G**

**Fig. 2 Contrast-enhanced CT, Before and After Treatment.**

**(A-B)  Pre-treatment axial CT images** show a massive infiltrative mass **(*)** encasing mesenteric vessels and bowel loops, exhibiting heterogeneous arterial phase enhancement (A) and persistent, near-homogeneous venous phase enhancement (B). **C  Post-treatment axial CT image** reveals marked regression of the mass with only residual patchy enhancement **(→)**. **(D-E) Pre-treatment volume-rendered (VR)** CT images depict the large hypervascular mass **(*)**, a markedly dilated superior mesenteric artery (SMA), and significant narrowing of the distal aorta **(→)**.  **(F-G) Post-treatment VR** images confirm mass regression**(*)** and mild improvement in the previously noted arterial narrowing(→).


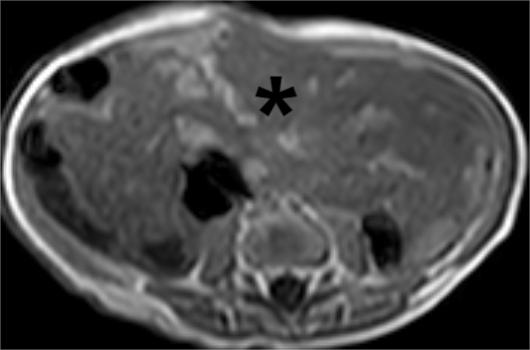

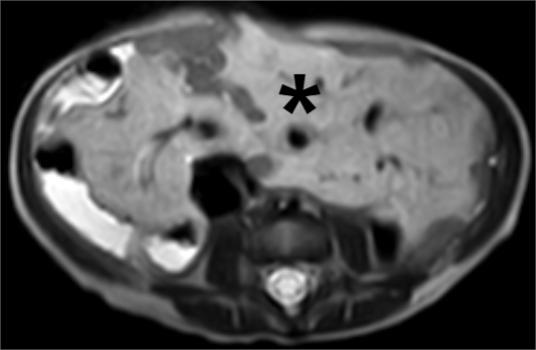

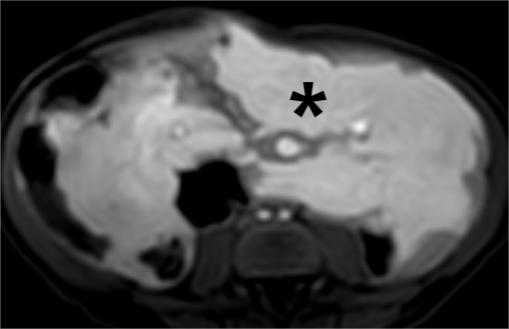


**3A 3B 3C**


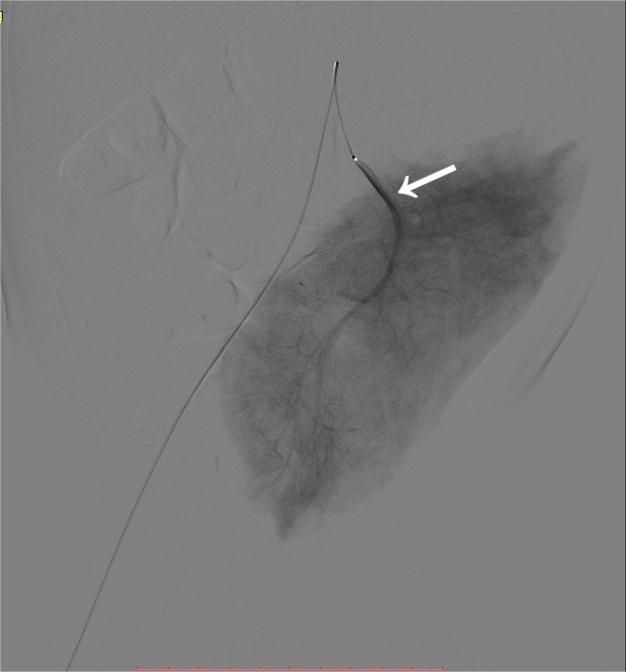

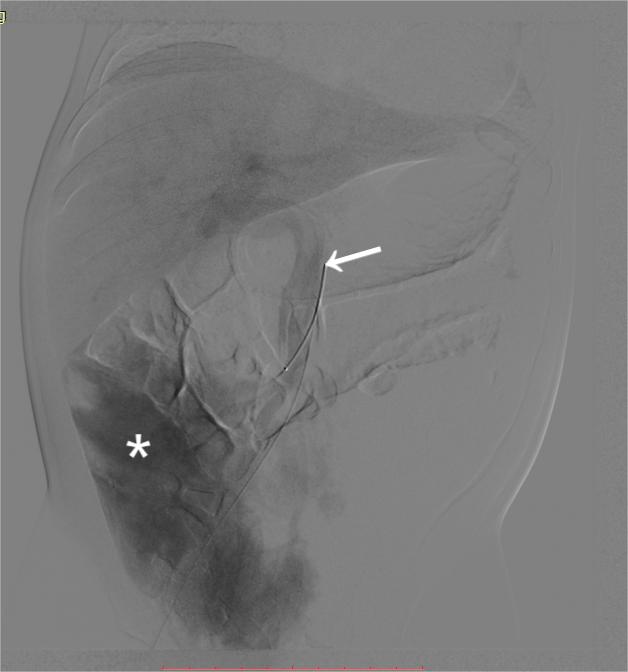

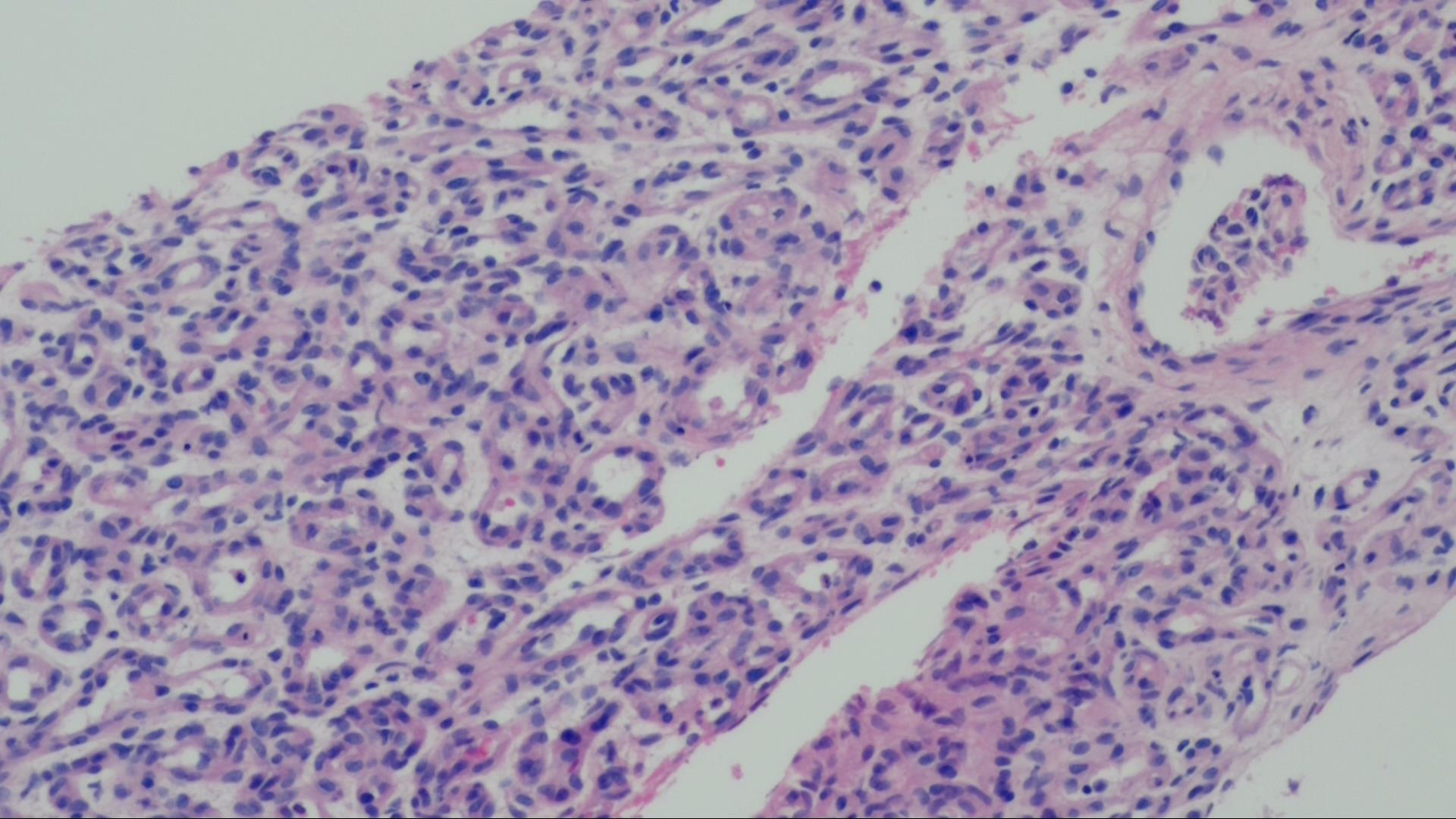


**3D 3E 3F**

**Fig. 3 Multimodality Characterization and Pathological Correlation of the Mass.
(A-C) MRI Features**  **A** Axial T1-weighted image shows the mass (*) as mildly hypointense.  **B**Axial T2-weighted fat-saturated image demonstrates marked T2 hyperintensity (*). **C**Axial contrast-enhanced T1-weighted image reveals progressive, intense enhancement (*). **(D-E) Digital Subtraction Angiography (DSA)** **D** Arterial phase frontal projection shows the mass supplied by hypertrophied branches of the superior mesenteric artery (SMA)**(→)**.**E** Substantive phase image reveals an intense, persistent tumor blush **(*)** with early opacification of the dilated portal venous system **(→)**, confirming high-flow shunting. **F** Photomicrograph (H&E stain) of the biopsy specimen shows proliferating capillaries lined by plump endothelial cells.
